# Wafer-Scale Periodic Poling of Thin-Film Lithium Niobate

**DOI:** 10.3390/ma17081720

**Published:** 2024-04-09

**Authors:** Mengwen Chen, Chenyu Wang, Xiao-Hui Tian, Jie Tang, Xiaowen Gu, Guang Qian, Kunpeng Jia, Hua-Ying Liu, Zhong Yan, Zhilin Ye, Zhijun Yin, Shi-Ning Zhu, Zhenda Xie

**Affiliations:** 1 National Laboratory of Solid State Microstructures, School of Electronic Science and Engineering, School of Physics, College of Engineering and Applied Sciences, Collaborative Innovation Center of Advanced Microstructures, Nanjing University, Nanjing 210093, China; mg20220198@smail.nju.edu.cn (M.C.); 602022220049@smail.nju.edu.cn (C.W.); jiakunpeng@nju.edu.cn (K.J.); liuhuaying@nju.edu.cn (H.-Y.L.); zhusn@nju.edu.cn (S.-N.Z.); 2 National Key Laboratory of Solid-State Microwave Devices and Circuits, Nanjing Electronic Devices Institute, Nanjing 210016, China; tangjieck@126.com (J.T.); gxw_tk@163.com (X.G.); chinaqgll@163.com (G.Q.); 3 School of Integrated Circuits, Nanjing University of Information Science and Technology, Nanjing 210044, China; zhongyan@njust.edu.cn; 4 NanZhi Institute of Advanced Optoelectronic Integration Technology Co., Ltd., Nanjing 210018, China; yezhilin@ioptee.com (Z.Y.);

**Keywords:** thin-film lithium niobate, quasi-phase matching, electric field poling, wafer-scale fabrication

## Abstract

Periodically poled lithium niobate on insulator (PPLNOI) offers an admirably promising platform for the advancement of nonlinear photonic integrated circuits (PICs). In this context, domain inversion engineering emerges as a key process to achieve efficient nonlinear conversion. However, periodic poling processing of thin-film lithium niobate has only been realized on the chip level, which significantly limits its applications in large-scale nonlinear photonic systems that necessitate the integration of multiple nonlinear components on a single chip with uniform performances. Here, we demonstrate a wafer-scale periodic poling technique on a 4-inch LNOI wafer with high fidelity. The reversal lengths span from 0.5 to 10.17 mm, encompassing an area of ~1 cm^2^ with periods ranging from 4.38 to 5.51 μm. Efficient poling was achieved with a single manipulation, benefiting from the targeted grouped electrode pads and adaptable comb line widths in our experiment. As a result, domain inversion is ultimately implemented across the entire wafer with a 100% success rate and 98% high-quality rate on average, showcasing high throughput and stability, which is fundamentally scalable and highly cost-effective in contrast to traditional size-restricted chiplet-level poling. Our study holds significant promise to dramatically promote ultra-high performance to a broad spectrum of applications, including optical communications, photonic neural networks, and quantum photonics.

## 1. Introduction

Lithium niobate (LiNbO_3_) has been exploited as an attractive platform of integrated photonics since it features exceptional material characteristics, for example, excellent nonlinear-optic ($\chi^{\left(2\right)}$ and $\chi^{\left(3\right)}$), electro-optic (EO), acoustic-optic, thermo-optic, piezoelectric and pyroelectric effects, as well as its wide transparency window (0.4~5 μm) [1,2]. Especially, LiNbO_3_ surpasses its counterparts in PICs such as Si and Si_3_N_4_ in its second-order optical nonlinearity. The large second-order nonlinear coefficient ($d_{33}$ = −27.2 pm/V) [3] along with low propagation loss makes LiNbO_3_ a preeminent platform for highly efficient frequency conversion processes. As an emerging technology, a lithium niobate on insulator (LNOI) structure is well suited for chip-scale nonlinear device integration under intensive study. Benefiting from a thin-film structure (300~900 nm) and large refractive index contrast, LNOI enhances the optical field overlap via tight confinement in a submicron scale with the flexibility of nanofabrication beyond a mere inheritance from LiNbO_3_ crystal [4,5,6,7].

Phase matching is essential for the efficient operation of $\chi^{\left(2\right)}$-based devices, facilitating a positive net transfer of energy from the pump frequency to the signal and idler frequencies or vice versa. Quasi-phase matching (QPM) is the predominant method used, achieving this energy transfer by artificially creating a periodic structure in the $\chi^{\left(2\right)}$-medium. To put it specifically in this paper, QPM modulates/reverses the nonlinear coefficient every coherence length. Experimentally, periodic poling (PP) technique assisted with a high electric field is adopted for periodic domain reversal due to the ferroelectricity of LiNbO_3_. Nowadays, waveguide etching has been popularized and matured for device fabrication with high performance. Diversified PPLNOI devices have thrived with promising applications in on-chip classical frequency conversion processes [8,9,10,11,12,13,14,15] and quantum light sources [8,16,17,18,19,20,21]. However, while these devices demonstrate impressive merits, the majority of them, utilized in scientific investigations, have been manufactured on a chip scale in laboratories [22]. In anticipation of mass production, large-scale fabrication methods are in urgent need to boost industrial applications in PICs [4]. The success in wafer-scale low-loss waveguides [15,17] pioneered foundry-compatible methods with LNOI akin to those used for silicon on insulator (SOI), and some devices, like EO modulators [18,19], have been realized in LNOI with wafer-scale photolithography. In principle, most of the LNOI devices can be fabricated using high-throughput photolithography. The PP technique, nonetheless, has never been endeavored in wafer scales, hampering the promotion of PPLNOI from lab to fab.

In this paper, we report wafer-scale PP for the first time to the best of our knowledge. We introduced specialized electrode designs, uniquely tailored for mass poling, that facilitate high throughput, efficiency, and stability unparalleled by traditional methods. Composed of comb-like positive electrodes and flat negative electrodes, 70 nm thick Cr plus Au was patterned on the 600 nm thick x-cut LNOI wafer with photolithography. Being adaptable for mass production, the comb widths were varied with their corresponding periods, and the pads were grouped by polarity within each block. By this means, the periodic domain structure in the whole wafer can be fabricated by applying a consistent voltage waveform with as few operations as possible. High-quality poling was confirmed via characterization by confocal second-harmonic microscopy [23,24], exhibiting 50% duty cycles, sufficient effective poling areas, thin domain walls, switching across the whole film thickness, and overall uniformity along the *z*- and *y*-axes. As a result, reversal lengths span from 0.5 to 10.17 mm across the wafer and a maximum area of ~1 cm^2^ can be poled with a single manipulation with periods spanning from 4.38 to 5.51 μm, suitable for potential $\chi^{\left(2\right)}$ applications in telecommunication bands. Zooming out to the entire wafer, we obtained uniform and high-fidelity poling results with a 100% success rate and an average 98% high-quality rate over all 21 blocks. This groundbreaking approach not only outperforms traditional chip-scale proofs of concept but also paves the way for the mass manufacturing of PPLNOI devices, marking a significant leap toward industrial-scale production. Our method, ready for application in a multitude of nonlinear processes for multifunctional complex PICs, represents a substantial advancement in the field, offering a scalable and efficient solution to the challenges previously faced by researchers and manufacturers alike.

## 2. Theory

Phase matching is always a crucial concern in nonlinear optical devices because sustained accumulation of frequency shifting is necessary for practical functions. A phase matching condition is commonly satisfied by the QPM process up to date. Taking LiNbO_3_ as a specific case, the largest nonlinear coefficient $d_{33}$ is periodically reversed every coherence length. Given the ferroelectricity of LiNbO_3_, the PP technique has been widely adopted for domain reversal in practice since early success in PP experiments in 1990s [25,26,27,28]. Throughout the past three decades, PP techniques have matured contemporarily with the advances of LiNbO_3_ platform from bulk to thin film [29,30]. The blooming PPLNOI devices have enabled frequency conversion processes, including second-harmonic generation (SHG) [8,31,32,33], sum-frequency generation (SFG) [16,34], difference-frequency generation (DFG) [9,10,11], and optical parametric oscillation (OPO) [12,13,14,35] with high efficiencies. In this section, we are going to talk about the QPM theory and construction in thin-film LiNbO_3_ and the most popular technical realization of it PP.

### 2.1. QPM

Theoretical discussion is a prerequisite for understanding the phase matching problem. In a $\chi^{\left(2\right)}$-mediated three-wave mixing process, energy conservation is satisfied:

(1) $$\omega_{1}{=\omega }_{2\;}{+\omega }_{3}$$

Meanwhile, there is the phase mismatch:

(2) $$\Delta {k=k}_{1\;}{-\;k}_{2\;}{-\;k}_{3}\;$$

where the wave vectors

(3) $$k_{i}=k_{i}e_{i}=\frac{n_{\mathrm{eff}}{(\omega }_{i}{)\omega }_{i}}{c}e_{i},\;i=1,\;2,\;3$$

where $n_{\mathrm{eff}}{(\omega }_{i})$ is the effective refractive index of a given mode with angular frequency $\omega_{i}$, and $e_{i}$ is the unit vector along the beam propagation direction. The nonlinear frequency conversion can be accumulated only when the phase is matched. That being said,

(4) Δk=0

We restrict ourselves in this paper to the waveguide configuration in that the wave vectors are collinear; hence, the momentum relations can be expressed in the scalar form.

The transparency window generally lies in one of the normal dispersion intervals ($\frac{{dn}_{\mathrm{eff}}}{d\omega }\;>\;0$) of optical media. In other words, $n_{\mathrm{eff}}{(\omega }_{1}{)\;>\;n}_{\mathrm{eff}}{(\omega }_{2})\;\geq {\;n}_{\mathrm{eff}}{(\omega }_{3})$ if $\omega_{1}{\;>\;\omega }_{2\;}\geq {\;\omega }_{3}$, so the phase cannot be matched inherently. In LNOI platforms, the phase matching condition is usually satisfied via the following techniques: birefringence phase matching (BPM), modal phase matching (MPM), and QPM. In a BPM and MPM process, the inequality chain is broken in a way that beams with different polarization and spatial modes are involved, respectively. To elaborate further, the incident angle needs adjusting about the optical axis elaborately, and both horizontally and vertically polarized beams must be engaged. MPM, otherwise, involves different spatial modes in a meticulously selected geometry, sacrificing mode overlaps. Such a rigorous geometry may not even exist in many nonlinear processes or under certain engineered dispersion. To sum up, both schemes are limited against exploiting the whole transparency window with an unconstrained geometry. The idea of QPM then dominates in LNOI devices because any nonlinear interactions across the transparency window can be noncritically phase matched in principle with suitable domain periods. Besides, QPM is flexible with arbitrary modes in any material-permitted sets of polarization configuration. Hence, it is an accompanied benefit that QPM qualifies as the optimal configuration for maximal frequency conversion in competition with BPM and MPM.

**Figure 1:**
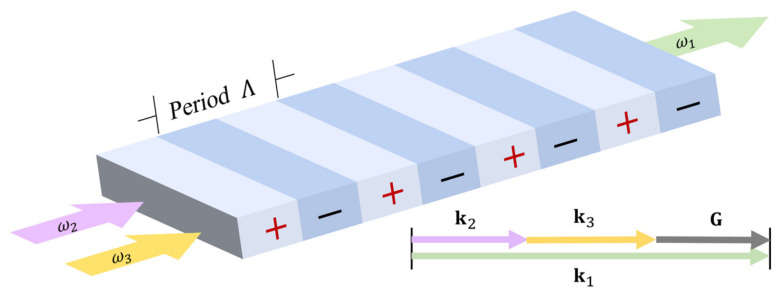
Schematic diagrams of QPM for a three-wave mixing process. The + and − represent the original and switched domains, respectively. Energy flows unidirectionally from $\omega_{2}+\omega_{3}$ to $\omega_{1}$ in a superlattice. The $\chi^{\left(2\right)}$ is modulated in a period Λ. The vector diagram illustrates the phase matching condition of QPM where the reciprocal lattice constant G makes up for the phase mismatch. Equation (6) states the expressions between G and Λ.

The phase matching condition of QPM is fulfilled via flipping domains every coherence length as shown in Figure 1. Such a periodic structure is called a superlattice, which provides a reciprocal lattice to compensate for the phase mismatch. The period Λ is reckoned as the superlattice constant. Under this scheme, Equation (2) is modified to be

(5) $$\Delta {k=k}_{1\;}{-\;k}_{2\;}{-\;k}_{3\;}-\;G\;$$

where the reciprocal lattice constant

(6) $$G=\frac{2\pi m}{\Lambda }b_{G}$$

where m∈ℤ is the order of QPM and $b_{G}$ is the unit reciprocal lattice vector. All vectors in Equation (5) are collinear in our configuration, so the phase mismatch can then be naturally simplified as a scalar expression. The numerical relation of Equation (5) is drawn in Figure 1 when Equation (4) is satisfied. Theoretically, Λ may be designed on demand to compensate for the phase mismatch in an arbitrary three-wave mixing process if it is structurally permitted in the transparency window. In our work, m=1 to reach the highest $\chi^{\left(2\right)}$ efficiency.

### 2.2. Periodic Poling

LiNbO_3_ is a ferroelectric with high spontaneous polarization $P_{s}$ pointing to the +*z*-axis. As indicated in Equation (6), QPM is realized via the fabrication of the superlattice structure shown in Figure 1. There exists a critical field strength in ferroelectrics called a coercive field ($E_{c}$) that a high pulsed field greater than $E_{c}$ from +*z* to −*z* must be applied to flip the local spontaneous polarization. The coercive field $E_{c}$ = 21 kV/mm for LiNbO_3_ crystal. There have been exhaustive studies on the ferroelectric domain inversion dynamics [21,36], which is acknowledged to be divided into four processes: nucleation of the reversed domain, longitudinal domain growth, lateral domain growth, and coalescence of local domains. Technically, the minimum electric field for domain inversion growth was found to be as high as 28.2 kV/mm, and evident lateral expansion can be observed only if an even greater field is applied [20].

The ways to pattern periodically switched domains in LiNbO_3_ mainly include femtosecond laser writing [37] and high voltage-assisted domain switching [32,38,39,40]. Although QPM was proposed early in 1962 [41], its practical feasibility was not proven until the 1980s. Since it was first experimentally accomplished in crystal growth [42], QPM was exhaustively applied in bulk LiNbO_3_ [43,44]. After that, weakly confined waveguides were developed [45,46,47] where the modes were confined to a few micrometers. PP technique on a mature LNOI platform came along afterward, and laser frequency conversion, quantum entangled light source, and nonlinear optical field control have been realized [4,32,48]. With the maturation and commercialization of thin-film LiNbO_3_, on-chip LiNbO_3_ devices are expected to maximize the nonlinear conversion efficiency with the superiority of miniaturization and low power consumption. The aforementioned discussions have highlighted flexibility, high conversion efficiency, and accessibility in experiments by applying QPM to LNOI.

## 3. Design and Experiment

### 3.1. Design of Electrodes

In the context of QPM devices on LNOI platforms, the intricacies of electrode design play a pivotal role in achieving optimal domain inversion. Navigated by PP dynamics, we now delve into the experimental consideration of wafer-scale poling on x-cut LNOI. In bulk crystals, the period is usually as large as 10~30 µm so that sufficient lateral space can be left for the electric field to diminish drastically along the lateral direction. Hence at a given voltage, the domain wall will no longer expand where the electric field is weaker than the minimum electric field for domain inversion. Such a phenomenon is called self-termination [49]. Different from the bulk case, the commonly adopted QPM periods are a few microns (usually 2.5~6.0 µm) in the LNOI platform. Self-termination comes after the coalescence of the reversed domains in LNOI. In addition to the challenge posed by small periods, the thin-film structure has also engendered more complex dynamics. The ensuing challenges make it imperative to compromise the design of the electrode and the pulse waveform for optimal duty cycles, switched domain quality (shapes, symmetry, uniformity, etc.), and reproducibility.

**Figure 2:**
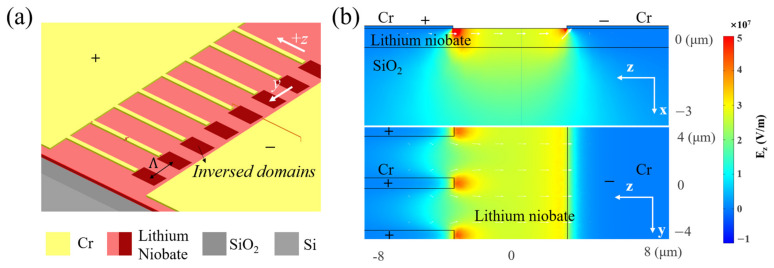
(**a**) Electrode configuration of x-cut LNOI. The electrodes are deposited on top of the LiNbO_3_ layer. The comb-shaped positive electrode is positioned on the +*z* side while the flat negative electrode is positioned on the −*z* side. Inverse domains are formed near the positive electrodes and grow longitudinally and laterally when pulses are applied. (**b**) $E_{z}$ distribution in both the *x*-*z* (a slice from the middle of an electrode comb) and *y*-*z* cross-section profile (a slice at a half thickness of LiNbO_3_) in the LNOI structure. Layer thickness: Cr and Au electrodes—70 nm thick, LiNbO_3_—600 nm thick, and SiO_2_—4.7 μm thick.

In our experiment, we designed an x-cut PPLNOI construction that adequately covers the nonlinear processes of S, C, and L bands (~100 nm bandwidth) for telecommunication applications. The electrode configuration of x-cut LNOI in our work is shown in Figure 2a. Since the domain inversion nucleates from the +*z*-axis [20], we used a comb-like positive electrode arranged in a periodic pattern on the +z side whose period is chosen in the 4 ~ 5.5 μm range, and a flat negative electrode on the −z side. The lengths of the positive electrode comb teeth are set as 20 μm, with the comb width about 1/5 of the period at a submicron level. The gap between the comb teeth tips of the positive electrode and the negative electrode is 6 μm. These shape parameters have been empirically examined as optimal for period poling on LNOI. The electrode layer consists of 40 nm thick Cr and 30 nm thick Au deposited on thin-film LiNbO_3_, which can ensure a considerable nucleation rate and sufficient material adhesion with the LiNbO_3_ thin film. The electric field simulation diagram for this configuration with a 600 nm thick LiNbO_3_ thin film is illustrated in Figure 2b. The electric field magnitude is greater than the minimum coercive field for switching across the poling area in both the *x*-*z* and *y*-*z* cross-section profiles, especially near the positive electrode. It is evident that this design of electrodes meets the requirements of poling electric field for domain reversal in entire thin-film LiNbO_3_.

### 3.2. Fabrication and Poling

We used a commercial 4-inch LNOI wafer from NANOLN composed of a 500 μm thick silicon substrate, a 4.7 μm thick thermal silicon dioxide (SiO_2_) insulating layer, and a 600 nm thick congruent thin-film LiNbO_3_. The thin film is fabricated by crystal ion slicing in combination with wafer bonding techniques generally known as “Smart Cut” [50]. Using photolithography technology combined with the metal lift-off process (photoresist AZ-5214E, developer AZ400K, and matching AZ stripper), we prepared a high-quality patterned electrode layer composed of 40 nm Cr and 30 nm Au for poling on a 4-inch wafer. The 0.15 and 0.5 μm Stepper photolithography machines are chosen to define electrode patterns for combs and pads in our work, respectively. Stepper makes patterning more rapid and economical with high throughput and is compatible with the tape-out process of photonic devices.

**Figure 3:**
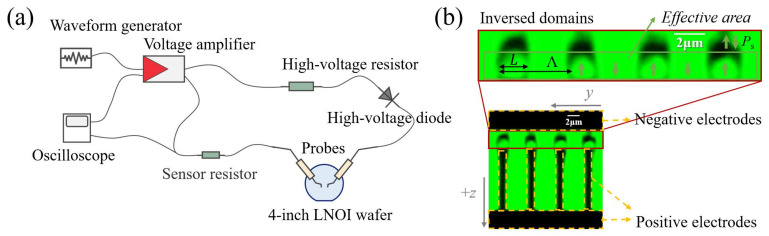
(**a**) The schematic diagram of the circuit setup for electric field poling of LNOI. (**b**) A typical confocal microscope image with electrode and domain structures poled with a typical set of poling parameters. The arrows represent the spontaneous polarization $P_{s}$ in each domain. The 2 μm scale bars are annotated.

The domain inversion was fabricated by high-voltage electric field poling introduced in Section 2.2. The wafer with the electrode layer was connected to the circuit shown in Figure 3a by a pair of probes. In order to prevent air breakdown caused by high electric field strength, a thin layer of silicone oil was dropped onto the electrode surface before poling. The voltage amplifier (Aigtek ATA-7020) amplifies the preset pulse generated from the arbitrary waveform generator (AWG) (Rigol DG4062) as the input of the circuit. We monitored both the amplified pulse signal and poling current as feedback in real time with an oscilloscope (Tektronix TDS 2024C). A high-voltage resistor and a diode were connected in series in the loop to protect the circuit against high current and backflow.

A pulse-shaped waveform was programmed in the AWG. In order to ensure the nucleation rate and domain spanning, a peak voltage that induces over 3$E_{c}$ was applied based on the poling period and electrode design at room temperature in our work [51], considering the higher coercive field of thin film than bulk LiNbO_3_ reported due to the bonding between the LiNbO_3_ thin-film layer and the SiO_2_ insulating layer and the external diffusion of Li^+^ caused by the annealing process in the LNOI fabrication process. A higher voltage can ensure the success rate of domain inversion; however, on the other hand, it incurs rapid lateral expansion of inverse domains, especially for small-period poling on LNOI. The ratio of the lateral domain width *L* to the period Λ is defined as the duty cycle. The extent of lateral expansion of the inverse domain determines the duty cycle, which, in turn, affects the nonlinear conversion efficiency. This competitive mechanism should be balanced for precision control of duty cycles where pulse area and repetitions are crucial parameters. High-efficiency nonlinear conversion requires high-quality poling domain structures, such as 50% duty cycles, sufficient effective poling areas, thin domain walls, inversion across the whole film thickness, and overall uniformity along the *z*- and *y*-axes. In light of this, suitable parameters in the experiment are crucial as they control the growth speed and final morphology of the domain structures. Additionally, a field lower than $E_{c}$ lasts after each poling cycle to prevent depolarization while not impacting the domain growth.

In our experiment, confocal second-harmonic microscopy was applied to examine the inverse domain structures. By irradiating the sample with a femtosecond laser, the second harmonic (SH) generated in the inverted domains will produce a phase shift compared with the non-inverted domains. This phase shift, due to destructive interference between adjacent domains of opposite polarization, results in diminished SH intensity near the domain walls. This technique allows for the rapid imaging of domain structures over a large field with diffraction-limited optical resolution. More important, such a microscopy is nondestructive to the sample. Figure 3b shows a typical post-poling image captured with confocal second-harmonic microscopy. The black regions enclosed by the yellow dashed lines are the positive and negative electrodes, and the red box highlights the periodically poled domain structures. Inverted domains grow longitudinally from the positive electrodes (+*z* axis) to the negative electrodes (−*z* axis), and lateral expansion occurs at the same time, leading to the formation of domain walls between adjacent inverse domains. The clear region with high brightness enclosed by the domain wall signifies a relatively uniform reversed domain. In this region, the domain is reversed over the entire LiNbO_3_ thin film thickness, inside which is an effective area for the nonlinear process.

## 4. Results and Discussion

### 4.1. Characterization of Inverse Domains: Single Devices

**Figure 4:**
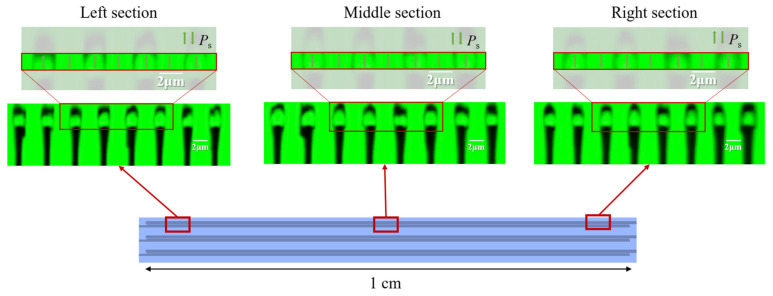
Confocal microscopy images of the left, middle, and right sections of a 1 cm long device. The poling period is around 4.38 μm here. The inverted domains in the red boxes are magnified for detail and the arrows represent the spontaneous polarization $P_{s}$ in each domain. The 2 μm scale bars are annotated.

Inverse domain qualities were characterized via confocal second-harmonic microscopy after suitable poling parameters had been managed. Only with adequate lengths of uniform poling can an attractive QPM device be practically applicable for nonlinear conversion processes. The poling quality of a device also determines its performance. When the optimized parameters are used, we could perform domain inversion over 1 cm along the *y*-axis on the wafer. This is long enough to cover the design length of current nonlinear devices. Figure 4 illustrates the schematic diagram of a poling device and the confocal microscopy images with periods around 4.38 μm. The images are taken from the left, middle, and right sections with an average duty cycle of 49.3% (σ = 1.5%), 50.6% (σ = 2.0%), and 50.8% (σ = 1.9%), respectively. This indicates that the required duty cycle interval (50 ± 5%) and good uniformity along the *y*-axis are maintained over the entire length. Bright inverted domains, thin domain walls, and sufficient effective areas with uniformity along the *z*-axis indicate high-quality poling. We define poling results with duty cycles of 50 ± 5% plus the properties mentioned above as high-quality results for statistics, and we have achieved periodic poling with a 100% high-quality rate by applying an external electric field in this device. Also, no depolarization phenomenon has been observed so far. This result in our work is almost indistinguishable from the samples using EBL lithography to define the electrode on the chiplet level [32,33,51].

### 4.2. Domain Inversion: Groups

**Figure 5:**
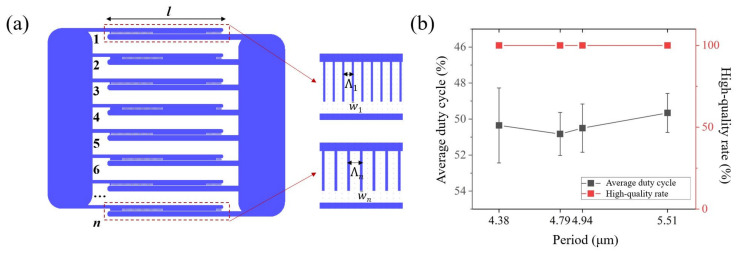
(**a**) The schematic illustration of the electrode group comprising 1 to *n* devices. Inset shows the *n*-th device with spanning length *l*, period $\Lambda_{n}$, and corresponding electrode line width $w_{n}$. (**b**) The data on the average duty cycles and high-quality rates of a group when the periods range from 4.38 μm to 5.51 μm.

In addition to obtaining high-quality results, another important focus on wafer-scale poling is high throughput, which requires minimizing the number of power-ups by moving the probes and maintaining the same poling parameters across the experiment. The devices designed on the entire wafer usually contain variations in periods or multiple periods. To maintain the same parameters, we adjusted the line widths of the positive electrode comb teeth, which are positively correlated with the lateral expansion speed of the inverted domains. This correlation allows for the application of the same high-voltage waveform to obtain inverted domains with different lateral broadening widths at the same time. That being said, the same duty cycle can be achieved concurrently in devices with different periods. Furthermore, by connecting the electrode pads into groups, a large area of the electrodes can be applied with the same external electric field at one time. This design of poling electrodes is shown in Figure 5a, and we define it as a *group*. A summary of the results of a group in our work is presented in Figure 5b. The area of 1 cm^2^ can be poled just once with a period interval over 1 μm (4.38~5.51 μm), resulting in a 100% high-quality rate. Repeated experiments were conducted on different groups, and the results demonstrate excellent stability, which evidences that this design can be extended to large-scale poling to greatly improve efficiency.

### 4.3. Domain Inversion: The Entire Wafer

**Figure 6:**
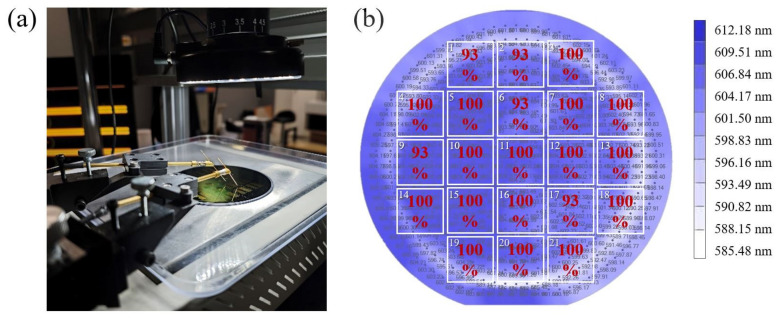
(**a**) The photograph of a periodically poled 4-inch LNOI wafer by the external high-voltage electric field in the experiment. (**b**) The schematic diagram is labeled by the high-quality rates of 21 blocks on the entire wafer. The background is a thickness fluctuation map of thin-film LiNbO_3_.

From the zoomed-out view, our experiment was conducted on a 4-inch wafer, and an efficient poling process was carried out based on the above-mentioned experimental design and methods. Figure 6a shows a photograph during poling. The positive and negative probes are contacted with the corresponding electrodes in each group. The wafer in our experiment was preset into 21 blocks labeled in Figure 6b, and the high-quality rate of each block with an area of 1.5 × 1.5 cm^2^ is summarized. The background is a thickness fluctuation map of thin-film LiNbO_3_ from NANOLN. We have achieved a 100% poling success rate of periods in the range of 4.38~5.51 μm and higher than 90% high-quality rate in each block, averaging an overall 98% high-quality rate. The electrode design and poling process in our work are proven to perform with high stability and uniformity on the entire 4-inch wafer scale and have a certain degree of insensitivity to variation in thickness. In the blocks that do not reach a 100% high-quality rate, a small part of the periodically poled domains have been fabricated with duty cycles outside the 50 ± 5% range but inside the 40~70% range. We conject such deviation as the initial parameters testing and slight changes in the conductivity of the poling electrodes. This deviation in the duty cycle does not cause a shift in the nonlinear conversion spectrum but simply has a small impact on the conversion efficiency [52], which can be finer poled locally after feedback from confocal second-harmonic microscopy.

## 5. Conclusions

In summary, we have designed and exhibited high-quality periodic poling processing tailored for the wafer-scale PPLNOI platform. Leveraging the principles of ferroelectric domain switching, we have optimized the poling pulse shape and repetition times for high-quality poling, ensuring 50% duty cycles with sufficient effective poling areas, thin domain walls, inversion across the whole film thickness, and overall uniformity along both the *z*- and *y*-axes. Comb line widths are meticulously adjusted to be compatible with the poling periods, and electrode pads within a block, corresponding to similar poling interval lengths, are strategically grouped to facilitate high-throughput and high-quality domain switching. Through careful electrode design and parameters optimization, the reversal lengths span from 0.5 to 10.17 mm and a maximum ~1 cm^2^ area can be poled just in a single operation. The resulting uniform and high-quality poling performance, with a period range from 4.38 to 5.51 μm, demonstrates a 100% success rate and a 98% high-quality rate on average. Our analysis indicates that the domain reversal quality achieved herein is comparable to, if not surpassing, those realized at the chiplet level. Our methodology underscores a mature poling scheme featuring high throughput, efficiency, and stability, markedly enhancing the production yield through a cost-efficient wafer-scale process. The foundry-compatible manufacturing approach has reliable process adaptability, which significantly advances the transition from chip-scale proofs of concept to industrial-scale production.

Looking forward, our work is poised for applications across a broad spectrum of nonlinear processes, such as frequency conversion, optical parametric amplification, and spontaneous parametric down-conversion. We aim to further expand the range of poling periods in our wafer-scale platform to support multiple quasi-phase matching processes to realize more efficient energy conversion across an expanded frequency range. This endeavor will enhance the integration of complex and diverse functionalities. Combined with the low-loss nanophotonic waveguide production via a reliable etching process, our large-scale poling technique aspires to fully harness the efficiency of thin-film LiNbO_3_ in $\chi^{\left(2\right)}$ processes. This envisioned integrated platform will be able to pave a feasible way for multifunctional and complex nonlinear PICs, finding its utility in applications such as optical telecommunications, photonic neural networks, and quantum photonics.

## Data Availability

The data presented in this study are available on request from the corresponding author. The data are not publicly available due to they are part of an ongoing study.
